# The short version of the ALR‐RSI scale is a valid and reproducible scale to evaluate psychological readiness to return to sport after ankle lateral reconstruction

**DOI:** 10.1002/jeo2.70160

**Published:** 2025-02-17

**Authors:** Alizée Mahieu, Mohamad K. Moussa, Eugénie Valentin, Ronny Lopes, Alexandre Hardy

**Affiliations:** ^1^ Clinique du sport Paris France; ^2^ Department of Orthopaedic Surgery and Sports Medicine Centre Orthopédique Santy, FIFA Medical Centre of Excellence, Groupe Ramsay‐Generale de Sante, Hôpital Privé Jean Mermoz Lyon France

**Keywords:** ALR‐RSI, ankle, ankle ligament reconstruction, instability, psychological readiness, return to sport (RTS)

## Abstract

**Purpose:**

To develop and validate a short and mini version of the ALR‐RSI (Ankle Ligament Reconstruction‐Return to Sport after Injury) scale.

**Methods:**

The ALR‐RSI scale contains 12 items and was administered to 109 patients following arthroscopic anatomical lateral ankle reconstruction. The short (6‐item) and mini (3‐item) versions were developed using a systematic selection process to eliminate items based on their category, mean, standard deviation and pertinence. A second group of 75 patients participated in an analysis to validate the predictive value of these scores. These patients filled out all three ALR‐RSI versions 6 months after arthroscopic anatomical reconstruction of the lateral ankle to determine the predictive value for the return to sport (RTS) at 12 months. The predictive value was evaluated with receiver operating characteristic curves (area under the curve [AUC]).

**Results:**

The long version of the ALR‐RSI had a high internal consistency (Cronbach's *α* = 0.97), suggesting redundancy of certain items. A short version of 6 items was developed (Cronbach's *α* = 0.94). A mini version of 3 items was also developed which retained one key item from each category. Factorial analysis confirmed that only one factor explained 76% of the total variance in the mini version (Cronbach's *α* = 0.89). The scores of the three versions were higher in patients who returned to sport at the same pre‐injury level of play or better (*p* < 0.0001). Both versions were found to have a good predictive value for the RTS at 12 months, with comparable AUC values (full version AUC 0.70 [95% confidence interval; CI, 0.57–0.83]; short version AUC 0.72 [95% CI, 0.59–0.84]); mini version, AUC 0.73 [95% CI, 0.61–0.85].

**Conclusion:**

The shorter versions (6 and 3 items) of the ALR‐RSI may be used to predict the RTS at the pre‐injury level without affecting the psychometric characteristics of the long score.

**Level of Evidence:**

Level II prospective cohort study.

**Abbreviations**
ACL: anterior cruciate ligament
ALR‐RSI: Ankle Ligament Reconstruction‐Return to Sport after Injury
ATFL: anterior talofibular ligament
AUC: area under the curve
CAI: chronic ankle instability
CFL: calcaneofibular ligament
PROMS: Patient‐Reported Outcome Measures
ROC: receiver operating characteristic
RTS: return to sport

## INTRODUCTION

Chronic ankle instability (CAI) in athletes develops frequently after lateral ankle sprains. Surgical treatment by arthroscopic anatomical lateral ankle reconstruction (anterior talofibular ligament [ATFL] and calcaneofibular ligament [CFL]) is proposed to these patients. The goal of treatment for most athletes is to return to their pre‐injury level of sport participation. The causes of a failure to return to sport (RTS) after surgery may be both physical and psychological [3, 18]. An international consensus has emphasized the importance of including self‐reported patient questionnaires on ankle function [15, 26]. The athlete's feelings, preconceived ideas, fear, anxiety, confidence in the injured limb and capacity to perform, influence his/her psychological readiness to RTS [5]. Evaluation of these elements is a key to determine the RTS and psychological scales have been developed for their measurement [8, 21, 25, 27].

The Ankle Ligament Reconstruction‐Return to Sport after Injury (ALR‐RSI) (Annex S1), published in 2020, is a valid, reliable, reproducible score to determine a patient's psychological readiness to return to their pre‐injury sport following ankle reconstruction [23]. This is a unidimensional scale with 12 items that measures three categories: emotions (5 items), performance confidence (5 items) and risk appraisal (2 items). Each item is measured on a scale of 0–10 and the total score is determined by adding the values of the 12 responses and then dividing the results by 1.2 to obtain a percentage. High scores correspond to a positive psychological response [15]. The ALR‐RSI score has recently been validated in patients with CAI who underwent a modified Broström–Gould procedure [17] and Achilles tendon repair [20], and it has been translated into and validated in French [1] and Spanish [13]. The internal consistency reported in these studies was often high (more than 0.9), suggesting redundancy in the questions.

With the development of PROMS (Patient‐Reported Outcome Measures), patients may find themselves having to respond to numerous questionnaires, which is time consuming and can negatively influence observance and follow‐up [28]. In addition, questions that may seem redundant require more time and thought to answer correctly. For these reasons, the authors of the ACL‐RSI scale developed a short version with six valid, reliable items and equivalent psychometric properties [25]. Reducing the length of these questionnaires without losing their psychometric properties can improve patient adherence and streamline clinical assessments. The goal of this study was to reduce the number of questions in the ALR‐RSI scale and determine its predictive value and discriminant capacity as well as the internal consistency of the short version compared to the original version. The aim was to offer the shortest version while maintaining the same psychometric properties. For this reason, a stepwise approach was used, starting with the validation of a 6‐item score. If redundancy was identified, a shorter 3‐item version was developed and validated to ensure efficiency without compromising accuracy.

The hypothesis was that the short (6‐item) and mini (3‐item) versions could be developed with the same psychometric properties as the long version.

## MATERIALS AND METHODS

### Patients

This study received approval from the local institutional review board (Ref: COS‐RGDS‐2021‐09‐001‐A). Written informed consent was obtained directly from patients.

**Figure 1:**
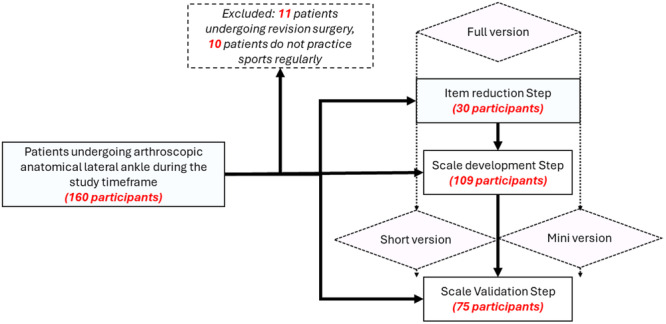
Diagram illustrating the process of developing and validating the short and mini versions of the ALR‐RSI scale.

A total of 109 patients (57 men, 52 women, mean age 33.1 ± 12.7 years old) who underwent arthroscopic anatomical lateral ankle (ATFL, CFL) reconstruction with a hamstring tendon graft within the last 12 months participated in shortening the scale in this study (Figure 1).

A second group of 75 patients who underwent arthroscopic anatomical lateral ankle (ATFL, CFL) reconstruction (38 men, 37 women, mean age 32.8 ± 13.7 years old) using the same technique participated in validating the predictive value in this study (Figure 1).

All patients were treated by the two surgeons specialized in sports surgery (A. H. and R. L.). Indication for surgery was based on patients experiencing pain and instability symptoms despite undergoing an initial course of conservative management. The reconstruction was offered for patients with Stages 3 and 4 (thin or absent ATFL) according to the French Society of Arthroscopy. The type and level of sports of the patients included are represented in Table 1.

**Table 1:** Baseline characteristics.

Baseline characteristics	*N* = 109
Age, mean (SD)	33.1 (12.7)
Gender, *N* (%)	
F	52 (47.7%)
M	57 (52.3%)
Level of sport, *N* (%)	
Competition	46 (42.2%)
Occasional leisure	16 (14.7%)
Regular leisure	32 (29.4%)
Professional	15 (13.8%)
Type of sport, *N* (%)	
Without pivot and without contact	29 (26.6%)
Pivot sport	35 (31.1%)
Pivot contact sport	45 (41.3%)

Acute cases were included to ensure a comprehensive representation of the population and to validate the score across various scenarios. Conservative treatment was carried out for 6 months minimum except for professional athletes who may benefit from treatment in the acute phase. All patients followed a uniform protocol: 3 weeks in a protective boot postsurgery, followed by physical therapy. RTS was allowed based Ankle GO score [14]. We have identified chronic and acute patients. There were *N* = 101 chronic patients (92.6%) versus *N* = 8 acute patients (7.3%). Chronic patients were defined as those in whom the delay between the start date of symptoms and the operation date was more than 6 months [9].

Patients were eligible for both groups if it was their first operation of the lateral ankle. All patients in this study were over 18 years old.

Patients were excluded if they had to undergo a second intervention within 12 months after the initial reconstruction or if they had another medical reason that prevented them from practicing their pre‐injury sport (e.g., pregnancy).

Weight‐bearing was allowed on postoperative day 1 depending on the patient's reaction. A walking boot was worn for 21 days. Running was permitted after 8 weeks as long as the patient recovered mobility comparable to the preoperative mobility. This was validated with 10 painless hops. Changing direction was then gradually reintroduced depending on the patient's reaction and the Ankle GO score [16].

#### Outcome measures and endpoints

The primary outcome measures of the study were the ALR‐RSI score and RTS. The primary endpoint of the study was the short version of the ALR‐RSI (6‐item). The secondary endpoint was the mini version of ALR‐RSI score (3‐item).

### Procedures and analyses

Approval was obtained from an ethics committee and patients provided written informed consent.

#### Item eligibility assessment

Before reducing the ALR‐RSI scale, an additional 30 patients who had undergone ankle reconstruction surgery in the last 12 months and who were, in terms of demographics, statistically similar to the cohort used for item reduction and validation, rated the importance of each item on a scale of 1–3 (1 = *unimportant*, 2 = *somewhat important*, 3 = *very important*) (Figure 1). To be eligible for inclusion in the shortened ALR‐RSI scale, items had to have a mean relevance score of at least 2.0 and a rating of at least ‘somewhat important’ by at least two‐thirds of the patients.

#### Item reduction and validation process

Participants in the scale reduction groups completed the 12‐item ALR‐RSI and indicated whether they had returned to sport and at what level (No RTS, RTS at a lower level, RTS at higher or same level).

All items were divided into three groups according to the classification by Webster and Feller [24] which includes emotions, confidence in performance and risk appraisal. When developing the short‐6‐item version and the mini‐3‐item version, it was essential to keep at least one question from each of the categories. Items for the short‐ and mini versions were included based on the following criteria [6]:

1/Items with a mean response score as close to 50/100 as possible.

2/Items with the highest standard deviation (SD).

3/Items with a mean relevance score of at least 2.

A correlation matrix was also used to compare the relationship between items. Any redundant items were considered for deletion from the scale.

After the items for the short‐6‐item and the mini‐3‐item scales were selected, we performed a correlation analysis to assess whether the correlation between the versions was still strong despite the reduction process. Divergent validity was assessed by comparing the scores of patients with a RTS at the pre‐injury level, a RTS at a lower level and those with no RTS, using the Kruskal–Wallis test and the pairwise Wilcoxon test with Bonferroni correction as the post hoc test. All scores were reported at 100 to facilitate comparisons.

#### Predictive validation process

In the predictive validation group, participants completed the 12‐item ALR‐RSI scale 6 months after surgery and answered a RTS questionnaire at 12 months. RTS questionnaire was a simple question that can be answered as: I have a RTS at the pre‐injury level, I have a RTS at a lower level, I did not RTS. The predictive value of the long 12‐item, the short‐6‐item and the mini‐3‐item versions were assessed using receiver operating characteristic (ROC) curve statistics. Two predictive analyses were performed: one for a RTS at the pre‐injury level of play or better and another for no RTS.

### Scale reduction

#### Short‐6‐item version

The complete version of the ALR‐RSI scale has been shown to have high internal consistency (Cronbach's *α* = 0.97), which suggests the presence of redundancy.

Items 4 and 5 of the 5 items examining emotions associated with RTS, refer to the fear of reinjury. The means and the SDs of these items showed that patients responded to both in a very similar manner. Item 5 was kept in the short version because its mean was closer to 50 and the SD was higher. Item 2 of the 3 remaining questions was kept because its mean was closer to 50. The mean and SD of items 1 and 3 were very close. Item 1 was chosen because it was more important.

Items 6, 7 and 8 of the 5 items measuring performance confidence refer to confidence in the ankle during sports. Item 7 was used in the short version because the mean was close to 50 and its SD was higher. Items 9 and 10 described the patient's confidence in his/her ability to perform. The ‘relevance score’ of item 10 was below 2 (score relevance 1.97) thus, item 9 was used.

The importance of items 11 and 12, the 2 items in the risk appraisal category was similar. Item 12 was used because the mean was close to 50 and the SD was higher.

#### Mini‐3‐item version

Three items (mini version) were selected from the 6 items in the short‐6‐item version, thus one item from each category, to create a mini version based on the same criteria as the short version. Item 1 was chosen from the emotion category, because this was found to have the highest pertinence in the questionnaire, and it had the strongest correlation with items 2 (*r* = 0.68) and 5 (*r* = 0.76). Item 9 was selected in the performance category because it was closest to the mean, with a high SD and good pertinence. Item 12 was selected because it was the only remaining item in the risk appraisal category.

### Statistics

All items were rated on a scale of 1–100, with 10‐point increments. The mean score for each item was presented as the mean and SD.

The Spearman correlation coefficient was used to estimate the correlation between the ALR‐RSI and the Foot and Ankle Ability Measure (FAAM) [10]. This coefficient was considered as ‘weak’ if *r* < 0.5, as ‘moderate’ if 0.5 < *r* < 0.7 and as ‘strong’ if *r* > 0.7 [11].

Factor analysis was performed for each of the versions (full‐12‐item, short‐6‐item and mini‐3‐item), and the Cronbach alpha coefficient *α*, was used to evaluate internal consistency. A Cronbach's *α* value < 0.70 indicates low internal consistency, while >0.90 suggests redundancy.

We evaluated the presence of a correlation between the full‐12‐item version, the short‐6‐item and the mini‐3‐item versions with the Spearman test.

The validity of the long‐12‐item, short‐6‐item and mini‐3‐item versions to determine psychological readiness to RTS at the pre‐injury level or any level were evaluated and assessed by ROC curve statistics. In general, an area under the curve (AUC) of 0.5 suggests no discrimination, 0.7–0.8 is acceptable, 0.8–0.9 is excellent while >0.9 is considered outstanding. The Youden index was applied to calculate the optimal cutoff scores, which allowed for determining readiness to RTS at the same level as pre‐injury as well as at any level for each version.

In this study, a sample size of 109 participants produced a two‐sided 95% confidence interval (CI) with a width <0.05 when the estimated Spearman's rank correlation is >0.95. All statistical analyses were performed using R software version 4.2 and a *p*‐value of <0.05 was considered to be significant.

A power analysis was conducted to determine the required sample size for detecting an AUC of 0.75 with a significance level of 0.05 and a desired power of 0.80, using a *κ* value of 1.2. The analysis indicated that a minimum of 17 cases and 21 controls are needed to achieve the specified statistical power. We conducted a post hoc power analysis for an AUC of 0.77 with 109 participants (68 cases and 41 controls). Assuming a null hypothesis of AUC = 0.5, an *α* level of 0.05, and a two‐sided hypothesis test, we obtained a statistical power of 99% using the power.roc.test function in R. This indicates that the study was sufficiently powered to detect a significant difference from random classification.

## RESULTS

### Scale reduction

The means (SD) and ranges of each ALR‐RSI item are shown in Table 2 as well as the relevance of each item.

**Table 2:** Scores for individual items on the ALR‐RSI scale (*N* = 109).^a^

	Mean (SD)	Range	Relevance score^b^
**Emotions**			
***1. Do you think you will be able to practice your pre‐injury sport at the same level as before?***	68.6 (27.9)	0–100	2.80
**2. Do you think you might re‐injure your ankle if you return to sport?**	60.2 (26.2)	0–100	2.30
3. Are you worried about playing your pre‐injury sport again?	67.3 (31.1)	0–100	2.30
4. Do you think your ankle will be stable when you return to sports?	71.3 (25.2)	0–100	2.53
**5. Do you think you can play sports without worrying about your ankle?**	59.1 (30.7)	0–100	2.43
**Performance confidence**			
6. Are you afraid of re‐injuring your ankle when you play sports?	60.1 (27.3)	0–100	2.53
**7. Are you frustrated about having to take your ankle into consideration when you play sports?**	57.5 (32.7)	0–100	2.20
8. Do you think your ankle will hold up under pressure?	69.9 (25.1)	0–100	2.30
***9. Are you afraid of accidentally hurting yourself again when you are playing sports?***	55.8 (28.7)	0–100	2.30
10. Does the idea of having to have another operation or physical therapy prevent you from practicing your sport?	70.6 (30.7)	0–100	1.97
**Risk appraisal**			
11. Are you confident in your ability to play your sport well?	69.0 (28.0)	0–100	2.30
***12. Do you feel relaxed at the idea of practicing your sport?***	68.6 (28.9)	0–100	2.30

^a^ Items retained in the short version of the scale are shown in bold, items retained in the mini version of the scale are shown in bold and italic.

^b^ Patients (*n* = 30) were asked to provide a rating with respect to the item's importance (1 = *unimportant*, 2 = *somewhat important*, 3 = *very important*), and mean relevance scores were calculated from their responses.

#### Short‐6‐item version

The short version included 6 items: items 1, 2 and 5 (emotions), 7 and 9 (performance confidence) and item 12 (risk appraisal). Two items in the emotion category were deleted, 3 in the performance confidence category and 1 in the risk appraisal category.

The Cronbach *α* value was 0.94 for the short‐6‐item version, which suggested the presence of redundant items.

#### Mini‐3‐item version

Thus, the mini version included 3 items: items 1 (emotions), 9 (performance confidence) and 12. Three items were deleted from the emotion category, 5 from performance confidence and 1 from risk appraisal.

Factorial analysis confirmed the presence of one underlying factor to explain 76% of the total variance. The Cronbach *α* of the mini version (3 items) was 0.89.

Both the short version (6 items) and the mini version (3 items) were strongly correlated to the long version (*r* = 0.99 and *r* = 0.97) with *p* < 0.0001).

### Scale validation

Score validities are presented in Table 3.

**Table 3:** Comparative scores between 3 scales (full, short, mini) according to sport status.

	Group A = No return, *N* = 46 (42%)	Group B = Return to lower level, *N* = 27 (25%)	Group C = Return to same or higher level, *N* = 36 (33%)	*p*‐Value^a^	Comparison 2 to 2	*p*‐Value^b^
Full‐12‐items scale, mean (SD)	53.1 (27.2)	64.0 (21.8)	80.4 (15.1)	<0.0001	A vs. B	0.38
					A vs. C	0.0001
					B vs. C	0.01
Short‐6‐items scale, mean (SD)	50.1 (27.0)	60.7 (22.4)	77.1 (17.1)	<0.0001	A vs. B	0.39
					A vs. C	0.0001
					B vs. C	0.02
Mini‐3‐items scale, mean (SD)	52.7 (27.5)	63.3 (22.4)	80.0 (16.3)	<0.0001	A vs. B	0.36
					A vs. C	0.0001
					B vs. C	0.009

a Kruskal–Wallis test.

b Pairwise Wilcoxon tests with Bonferroni correction.

The RTS in the population selected for score validation was 58%, all levels of play included (25% at a lower level of play and 33% at the same level or better).

#### Divergent validity

There was a significant difference among the scores in the three groups (A = No RTS, B = RTS at a lower level and C = RTS at same or higher level) (*p* < 0.0001).

There was a significant difference between patients with no RTS (Group A) and those whose RTS was at the same or a higher level of play (Group C) with a higher score in Group C for all versions (*p* < 0.0001).

In the same way, there was a significant difference between patients with a RTS at a lower level of play (Group B) and those with a RTS at the same or a higher level (Group C) with a higher score in Group C in the long (*p* = 0.01), short (*p* = 0.02) and mini (*p* = 0.009) versions.

On the other hand, there was no difference in any of the three versions of the ALR‐RSI between patients who did not RTS (Group A) and those with a RTS at a lower than the pre‐injury level (Group B) (*p* = 0.38 long version, *p* = 0.39 short version, *p* = 0.36 mini version).

#### Discriminant capacity

The scores of the three ALR‐RSI versions were significantly discriminant for the RTS at the pre‐injury level compared to the other groups (no RTS and RTS at a lower level of play).

### External validity

The external validity is presented in the Table 3. There was a strong correlation between ALR‐RSI short and ALR‐RSI mini scores and FAAM with *r* > 0.5.

#### Predictive value for the RTS at pre‐injury level or better

**Figure 2:**
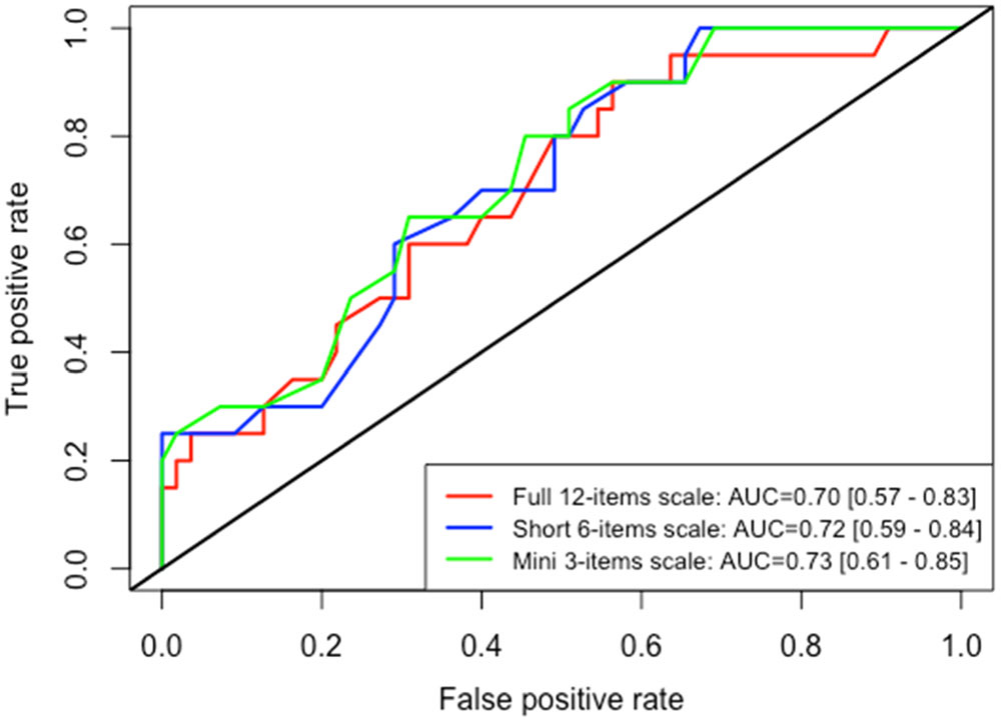
Receiver operating characteristic curve for predicting return to pre‐injury level of sport or higher. AUC, area under the curve.

The ROC curves are presented in Figure 2.

Twenty patients (25%) had a RTS at the pre‐injury level or better at postoperative month 12. The predictive value of the long, short and mini versions of the ALR‐RSI scale were acceptable for the RTS at the pre‐injury level (long version AUC 0.70; 95% CI, 0.57–0.83, short version AUC 0.72; 95% CI, 0.59–0.84, mini version AUC 0.73; 95% CI, 0.61–0.85). A reduction in the number of items did not decrease the predictive value.

The Youden index for the three versions (long, short and mini), was 0.34, 0.33 and 0.35, respectively corresponding to the scores of 68, 77 and 77 points, respectively with a sensitivity of 60%–65% and a specificity of 69%–71%.

#### Predictive value for RTS to any level

**Figure 3:**
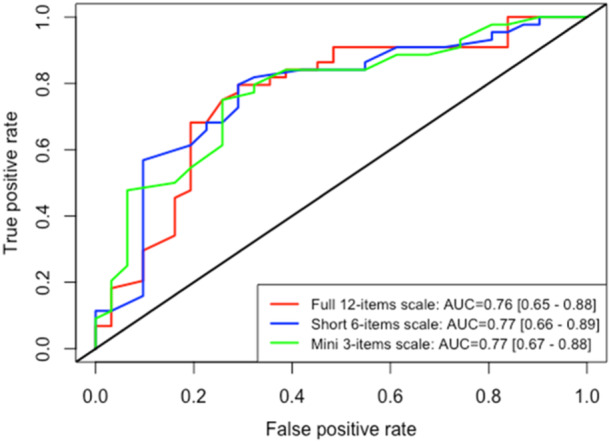
Receiver operating characteristic curve for three scales to predict the return to sport at all levels. AUC, area under the curve.

The ROC curves are presented in Figure 3.

There were 40 patients (45%) with no RTS at postoperative month 12.

The predictive value of the long, short and mini versions of the ALR‐RSI scale were acceptable for the RTS at the pre‐injury level; the AUCs were nearly identical (long version AUC 0.76; 95% CI, 0.65–0.88, short version AUC 0.77; 95% CI, 0.66–0.89, mini version AUC 0.77; 95% CI, 0.67–0.88).

The Youden index of the three versions (long, short and mini), was 0.51, 0.50 and 0.49, respectively with scores of 59, 58 and 67 points, respectively with a sensitivity of 7580% and specificity of 71%–74%.

## DISCUSSION

The main result of this study was that it is possible to develop short (6‐item) and mini (3‐item) versions of the ALR‐RSI score while preserving the psychometric properties of the long version. This is significant because it increases adherence to these questionnaires, especially when used alongside a battery of tests necessary to decide on RTS [19]. For instance, in the context of the Ankle‐GO score to predict RTS, where the long version of the ALR‐RSI is used in addition to other neuromuscular tests, having shorter versions can streamline the evaluation process. Similar to the study that shortened the ACL‐RSI score to 6 items, this study presents validated short and mini scores that maintain the integrity and predictive power of the original scale, but in a more practical and efficient format. These simplified questionnaires are less burdensome for patients, leading to higher completion rates while maintaining reliable data collection. Clinicians can quickly and efficiently assess psychological readiness without compromising the quality of the evaluation, facilitating better patient management.

Our study shows that the discriminant capacity of the short and mini versions of the ALR‐RSI are good for the RTS compared to no RTS both at postoperative months 6 and 12. This good discriminant capacity at two important points in time confirms the practical use of the short versions of the ALR‐RSI score for long‐term patient follow‐up.

The results of a previous study by Clanton et al. were similar; they confirmed the importance of psychological evaluations for the prediction of RTS, and noted that factors such as stress, loss of confidence, fear and apprehension can negatively influence rehabilitation. They suggested associating function tests and the evaluation of psychological factors with postoperative or postinjury rehabilitation protocols [4]. Our short or mini versions are an ideal tool in these cases, allowing physical therapists to follow the psychological progress of their patients on a day‐to‐day basis, while limiting the number of questions asked. This approach is supported by academic societies such as the American College of Sports Medicine, American Academy of Family Physicians and the American Academy of Orthopaedic Surgeons and confirms the importance of psychological aspects of sports injuries such as stress, pain management, communication or psychological readaptation [2].

The internal consistency and reliability of the short version are high, making it possible to predict a RTS compared to no RTS. Because the consistency was too high, suggesting possible redundancy, a mini version was developed. The ROC curves of the different versions of the ALR‐RSI at 6 months showed an identical predictive value for a RTS at 12 months. With thresholds of 59 for the short version of the ALR‐RSI and 58 for the mini version, both versions have a moderate sensitivity and specificity (75%–80% and 71%–74%, respectively).

This is especially important from a practical point of view, because of the absence of a consensus on specific criteria for an optimal time for RTS. The study by Wikstrom et al. discussed this and also the correlation between the results of tests to determine RTS and elements reported in patients with CAI, such as self‐confidence, management of fear or of another injury [7, 22]. Picot et al. also made an important contribution by developing and validating the Ankle‐GO score which includes objective, subjective and psychological criteria based on the long ALR‐RSI score to predict the RTS after ankle sprain. Because the short versions of the ALR‐RSI have preserved the key psychometric properties, they could effectively replace the long Ankle‐GO score, simplifying the test while maintaining its integrity and predictive value [16].

The short ALR‐RSI was developed using the same model as the long ACL‐RSI (2018). It contains 6 out of the 12 items from the long ACL‐RSI, which has been shown to have high internal consistency and to be a valid tool to evaluate psychological readiness to RTS following anterior cruciate ligament (ACL) reconstruction [24]. Although we used the same methodology as Webster et al. our selected items were not the same. In particular, items 4 (emotions) and 11 (risk appraisal) selected by Webster were replaced by items 5 (emotion) and 12 (risk appraisal) in our score. This reinforces the methodology used in that article, based on the idea that each joint should be managed independently, and that simply applying one validated short score to another joint is not feasible. In other words, simply selecting the items chosen by Webster in the short ALR‐RSI and validating them in the ankle was not viable and would have limited scientific value.

It is interesting to note that authors of other shortened scores did not develop a mini version. Despite a Cronbach's *α* coefficient of 0.92 for the short ACL‐RSI by Webster et al. suggesting possible internal redundancy, the authors felt that it was essential to keep 6 items. They felt that it was crucial for healthcare professions to determine which self‐reported information provided by the patient was pertinent. In the same way, Ignacio Pasqualini et al. developed a short SI‐RSI (Shoulder Instability Return to Sport after Injury) scale, using the same model as the ACL‐RSI, to predict the RTS following Bankart‐type arthroscopic or open Latarjet‐type shoulder repairs [12]. These authors chose to reduce the scale to 5 items at the outset, to minimize the high Cronbach's *α* coefficient and avoid dual reduction and validation phases. This strategy resulted in a Cronbach's *α* coefficient of 0.86 for the short SI‐RSI, with an absence of redundancy and good internal consistency [12].

In our study, the redundancy of the items in the short ALR‐RSI score, shown by a Cronbach's *α* coefficient of 0.94, justified the development of a mini version of our score. The short and mini versions simplify what is required from the patient and save time in an environment with multiple PROMs. It is also more efficient for the clinician, who can obtain a reliable, rapid evaluation with three questions, and save a significant amount of time for both parties.

### Limitation

One of the limits of this study is the deletion of certain items in the questionnaire, which could result in a less complete evaluation of the patient's psychological status. However, the short and mini versions keep at least one question from each key evaluation category.

While this study focused on a specific cohort of patients undergoing arthroscopic anatomical lateral ankle reconstruction, primarily composed of sportsmen, the findings may have broader implications. However, sportsmen may have different psychological and motivational profiles compared to other populations, which may limit the generalizability of these findings to nonathlete populations or those with different injury types and rehabilitation contexts.

The size of the study population could also seem small; however, a power calculator was used to determine the adequate sample size before the study.

## CONCLUSION

Two equally effective short versions (short‐6‐item and mini‐3‐item score) were developed from the long ALR‐RSI scale by deleting redundant items. Although these shorter versions maintain internal validity, their performance in our population suggests only acceptable predictive capacity, with moderate sensitivity and specificity values.

Additionally, the external validity of these tests was moderate, as shown by their moderate to low correlation with the FAAM.

These shorter versions enhance clinical applicability by increasing patient adherence and providing quick, reliable assessments of psychological readiness to RTS.

## AUTHOR CONTRIBUTIONS

Alizée Mahieu, Mohamad K. Moussa and Alexandre Hardy contributed to the conception, design of the study and interpretation of results. Alizée Mahieu, Ronny Lopes and Alexandre Hardy contributed to the acquisition of patients and their data. Alizée Mahieu and Mohamad K. Moussa contributed to the review of the database and critical review of data. Alizée Mahieu, Mohamad K. Moussa, Eugénie Valentin, Ronny Lopes and Alexandre Hardy contributed to critical review of the manuscript. Eugénie Valentin performed the statistical analysis and drafting of tables and figures. Alizée Mahieu and Mohamad K. Moussa contributed to the original creation and implementation of clinical databases.

## CONFLICT OF INTEREST STATEMENT

The authors declare no conflicts of interest.

## ETHICS STATEMENT

This study involves human participants and was approved by the ethics committee. Participants gave informed consent to participate in the study/collection of data before taking part.

## PATIENT AND PUBLIC INVOLVEMENT

Patients and/or the public were not involved in the design, or conduct, or reporting, or dissemination plans of this research. Though feedback about the collection of information for the database is encouraged.

## Supporting information

Supporting information.

## Data Availability

Data are available upon reasonable request.
