# Supplementary figures and images for: The short version of the ALR‐RSI scale is a valid and reproducible scale to evaluate psychological readiness to return to sport after ankle lateral reconstruction
Source: J Exp Orthop. 2025 Feb 17;12(1):e70160. doi: 10.1002/jeo2.70160 (PMC11832589; doi:10.1002/jeo2.70160)

**ANNEXE 1**

**Ankle Ligament reconstruction-return to sport after injury (ALR-RSI)**


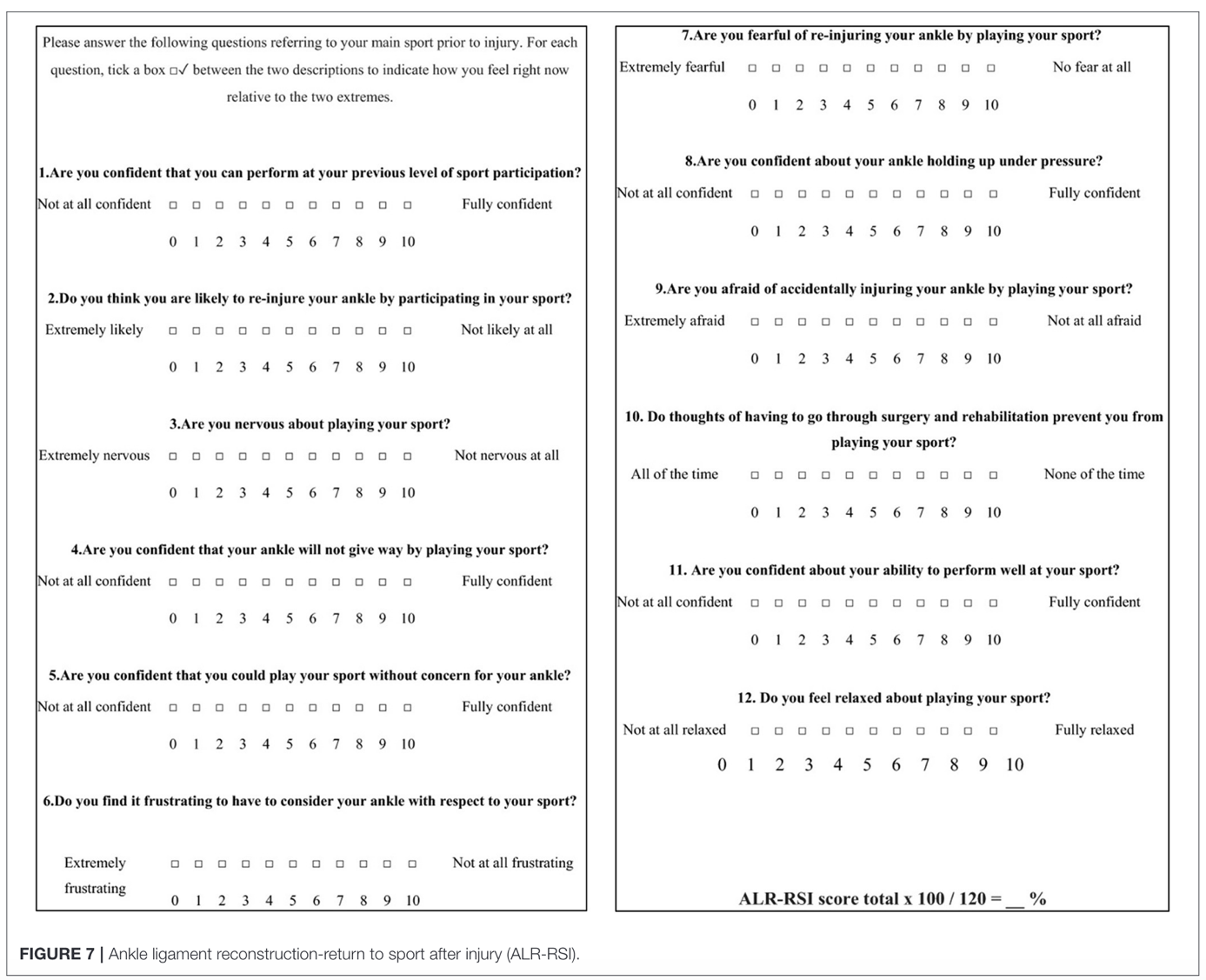

Supplement: Supplementary file 1 — Supporting information. [file JEO2-12-e70160-s001.docx]
